# Low handgrip strength is associated with diabetic foot disease in geriatric patients with type 2 diabetes

**DOI:** 10.55730/1300-0144.5532

**Published:** 2022-07-24

**Authors:** Eren İMRE, Neşe TERLİCAN KOÇAKGÖL, Erdi İMRE

**Affiliations:** 1 Department of Endocrinology and Metabolism, Dr. Ersin Arslan Education and Research Hospital, Gaziantep, Turkey; 2 Department of Orthopaedics and Traumatology, Abdulkadir Yüksel State Hospital, Gaziantep, Turkey

**Keywords:** Diabetes, foot, ulcer, handgrip, strength

## Abstract

**Background/aim:**

This study aims to reveal the prevalence of low handgrip strength in older patients with type 2 diabetes who have diabetic foot disease and to assess the association of handgrip strength with diabetic foot disease in older patients with type 2 diabetes.

**Materials and methods:**

Eighty-nine geriatric patients with diabetic foot ulcers and 69 patients without diabetic foot ulcers who presented to the endocrinology outpatient clinic between August 2020 and November 2021 were included in the study. The exclusion criteria were the usage of steroids, stroke-induced quadriplegia, myopathy, disability, hemodialysis treatment, type 1 diabetes, patients under 65 years of age, and history of malignancy. The information of drugs administered, demographic and clinical data were obtained from the patient files. The Wagner score was used to evaluate the severity of ulcers. A handgrip strength test was performed with a hand-held digital dynamometer. For females <16 kg (kilograms), for males <27 kg was accepted as low handgrip strength.

**Results:**

Forty-nine patients (55.1%) with diabetic foot ulcers and 25 (36.2%) patients without diabetic foot ulcers had low handgrip strength. There was a significant difference between two groups (p = 0.019). The patients with diabetic foot ulcers who had lower handgrip strength had higher rates of peripheral artery disease than patients with diabetic foot ulcers who had normal handgrip strength (p = 0.02 and p = 0.009, respectively). The patients with diabetic foot ulcers who had lower handgrip strength, had significantly higher rates of Wagner scores 4 and 5 and lower rates of Wagner scores 1 and 3 (p = 0.039).

**Conclusion:**

Older patients with type 2 diabetes and diabetic foot disease had a higher rate of low handgrip strength. Low handgrip strength was significantly associated with the occurrence of diabetic foot ulcers and directly correlated with Wagner score in geriatric patients with type 2 diabetes.

## 1. Introduction

Diabetic foot disease is the most common cause of nontraumatic lower-limb amputation internationally. It has been estimated that a lower limb is amputated every 30 seconds somewhere in the world as a result of diabetes, and the majority of these amputations are reported to occur after a foot ulcer [1–3]. It is also estimated that people with diabetes have a 19%–34% lifetime risk of occurrence of a foot ulcer [4,5], which is an important cause of death, morbidity, and cost to health systems and patients [1,6].

Grip strength is indispensable in daily activities, such as grasping, twisting, picking up, or lifting objects. It has been used as a marker of nutritional status and activities of daily living [7–9]. Grip strength is a measure of muscle function and it is one of the tests to assess sarcopenia [10]. As a consequence, grip strength has been suggested as a biomarker of aging [11]. It is a quick, inexpensive, and noninvasive test that can be performed easily. Its association with health outcomes including death, disability, and increased length of hospitalization has been extensively studied in older patients [12]. Furthermore, grip strength reflects variables such as nutritional status, and frailty [7, 13–15].

Recent research interestingly revealed the association between grip strength and wound healing [16]. Patients with low grip strength were 50% less likely to have their wounds healed compared with those with adequate grip strength when corrected for other factors [16]. There is a lack of information about the association between handgrip strength and diabetic foot disease. This study aims to reveal the prevalence of low handgrip strength in older patients with type 2 diabetes who have diabetic foot disease and to assess the association of handgrip strength with the existence and grade of diabetic foot disease in older patients who have type 2 diabetes.

## 2. Material and methods

### 2.1. Study design and patients

This single-center crosssectional observational study aimed to clarify the prevalence of low handgrip strength in type 2 diabetic geriatric patients with and without diabetic foot ulcers and evaluate the relationship between diabetic foot ulcers and handgrip strength. The study was conducted in the endocrinology outpatient clinics of Dr. Ersin Arslan Education and Research Hospital, Gaziantep, Turkey.

Patients with type 2 diabetes who presented to the endocrinology outpatient clinic between August 2020 and November 2021 were involved in the study. The patients are classified into two groups according to the presence of diabetic foot ulcers. Overall, one hundred and fifty-eight patients with type 2 diabetes (89 patients with diabetic foot ulcers and 69 patients without diabetic foot ulcers) were included in the study.

The exclusion criteria were as follows:

1. Patients under 65 years of age;
2. Type 1 diabetes;
3. Hemodialysis treatment;
4. The history of malignancy;
5. Patients using steroids and those with stroke-induced quadriplegia, myopathy, or mobility disability.

### 2.2 Clinical data

Demographic and clinical data were obtained from patient files recorded in the computerized data system of our hospital. Details about the presence of hypertension and hyperlipidemia, duration of diabetes, presence of microvascular and macrovascular complications, comorbidities, and medications used were recorded. Body mass index (BMI) was calculated based on height and weight. Anthropometric data recorded at examination and data of the fasting plasma glucose (FPG) and HbA1c values measured at our center were recorded and used as glycemic control parameters. Glomerular filtration rates (GFR) of patients were calculated using the modification of diet and renal disease (MDRD [mL/min/1.73m2]) equation (GFR = 186 × [Creatinine/88.4] −1.154 × [Age]−0.203 × [0.742 if female] × [1.210 if black] ).

The Wagner score (n = 5) was used to evaluate the severity of ulcers. The Wagner classification is the most commonly used to grade diabetic foot ulcers. The Wagner system was designed in 1976 by Meggitt and adjusted in 1981 by Wagner [15]. Wagner scores categorize DFU based on the ulcer depth into six grades (from G0 to G5).

### 2.3 Assessments of muscle strength

A handgrip strength test was performed to evaluate muscle strength with a hand-held Smedley digital dynamometer (Baseline 12-0286 Digital Smedley Dynamometer, Fabrication Enterprises Inc., Elmsford, NY, USA). Three measurements were taken 1 minute apart in each hand and the mean value of all measurements was used as the score for each patient. The measurement was applied according to the NHANES muscle strength/grip test procedure [17]. For females <16 kg (kilograms), for males <27 kg was accepted as low handgrip strength [10].

### 2.4. Ethical standards

The study protocol was approved by the Clinical Research and Ethical Committee of the Gaziantep Sanko University (Protocol 2020/04-06) and conducted at the Dr. Ersin Arslan Education and Research Hospital Endocrinology and Metabolism Outpatient Clinics on Harmonization Guidelines for Good Clinical Practice and the Declaration of Helsinki. All participants gave written informed consent.

### 2.5. Data analysis

All statistical analyses were performed using the statistical package SPSS (Statistical Package for the Social Sciences) v.22.0 (IBM Corp., Armonk, NY, USA), which was accessible from our network. Data were evaluated as mean (+/−) standard deviation for the continuous variables (FPG, weight, lipid profile), and as number (n) and percentage (%) values for categorical variables (sex, etc.). Normal distribution was tested using the Kolmogorov-Smirnov test. An independent sample t-test or Mann-Whitney U variance analysis test was used based on the assumption of normal distribution. Numerical data in dependent groups were compared using the dependent sample t-test or Wilcoxon test chosen per the normal distribution assumption. The chi-square test was used for comparing the categorical data. The correlation between low handgrip strength and numerical data (HbA1c, creatinine, fasting glucose level, etc.) was calculated using Spearman correlation analysis. The phi coefficient Cramer’s V is used to assess the association between two dichotomous categorical variables. The rank-biserial correlation coefficient, *r*_rb_, is used for dichotomous nominal data vs. rankings (ordinal). Stepwise proportional hazards multivariate regression was used to assess the association of putative baseline risk factors with the subsequent development of diabetic foot ulcers. A value of p less than 0.05 was considered statistically significant. Power analysis for the study was calculated using G-Power software, version 3.1.9.4 to calculate the effective sample size required for our study. The power of the study was calculated as 98% by considering the 5% error rate of the post hoc power analysis method based on the existence of low handgrip strength in both groups containing 158 subjects.

### 2.6. Biochemical measurements

Biochemical results measured in the last month were recorded from the patients’ files. Total cholesterol, high-density lipoprotein (HDL) cholesterol, triglycerides, and fasting plasma glucose were calculated by colorimetric-enzymatic methods (Siemens Advia System, Deerfield, IL, USA). HbA1C was measured by immune – inhibition on Advia 2400 chemistry system (Siemens Healtcare Diagnostics Inc, USA). Serum creatinine levels were measured using an ADVIA 1650 Chemistry system (Siemens, USA).

## 3. Results

### 3.1. Clinical characteristics and laboratory results of patients

The ‘mean +/− SD’ age of all of the type 2 diabetic patients was 70.13 ± 4.57 years, 84 (53.16%) patients were male and 74 (46.83%) were female. The duration of diabetes was 15.03 ± 7.22 (1–35) years. The ‘mean +/− SD’ values of BMI was 31.81 ± 5.93 kg/m2 and HbA1c levels were 9.63 ± 2.41%.

Clinical and laboratory characteristics of the patients according to the presence of diabetic foot ulcers are summarized in Table 1. Female sex was significantly less common in patients with diabetes who had diabetic foot ulcers (p = 0.047) and the duration of diabetes was significantly longer (p = 0.046). There were no significant differences between two groups, in terms of weight and body mass index.

### 3.2. Comorbidities

There were no significant differences between patients with type 2 diabetes who had and who did not have a diabetic foot ulcer, in terms of comorbidities (hypertension, hyperlipidemia, coronary artery disease, diabetic retinopathy) and habits of smoking (Table 1). However, peripheral artery disease and complaints of diabetic neuropathy were more common in the group of patients with diabetic foot disease (p = 0.004 and p = 0.005, respectively).

### 3.3. Medication use

There were no significant differences between two groups, in terms of the use of oral antidiabetic and anti-hypertensive drug profiles (Table 2). But the use of insulin treatment was more common in patients with type 2 diabetes who had diabetic foot ulcers (p = 0.004). Moreover, the use of statins was significantly more common in patients with type 2 diabetes who did not have diabetic foot ulcers (p = 0.007).

### 3.4. Muscle strength measurement

Of the 158 patients with handgrip measurement, 74 (46.8%) had low muscle strength. Forty-nine patients (55.1%) with diabetic foot ulcers and 25 (36.2%) patients without diabetic foot ulcers had low handgrip strength (p = 0.019).

Demographic and laboratory data of patients with diabetic foot ulcers classified as low and normal muscle strength are shown in Table 3. There was no significant difference between low or normal handgrip strength groups for age (0.070), sex (p = 0.462), duration of diabetes (p = 0.611). However, the body weight and body mass index were significantly lower in patients with diabetic foot ulcers (p = 0.032 and p = 0.001, respectively). The rates of hypertension, hyperlipidemia, ischemic heart disease, and the presence of complaints due to diabetic neuropathy were similar between two groups (Table 3). But patients with diabetic foot ulcers who had lower handgrip strength had higher rates of peripheral artery disease than patients with diabetic foot ulcers who had normal handgrip strength (p = 0.02 and p = 0.009, respectively).

The rates of insulin treatment, duration of insulin treatment, and total daily insulin doses were similar between patients with diabetic foot ulcers who had low and normal handgrip strength (p = 0.195, p = 0.504, and p = 0.355, respectively).

The levels of fasting plasma glucose, HbA1c, triglyceride, HDL and LDL cholesterols, urea, creatinine, and uric acid were similar between patients with diabetic foot ulcers who had lower and normal handgrip strength (Table 3).

Furthermore, patients with diabetic foot ulcers who had lower handgrip strength, had significantly higher rates of Wagner scores 4 and 5 and lower rates of Wagner scores 1 and 3 (p = 0.039, Table 3).

### 3.5. Correlation and regression analysis

There was a weak positive relationship between low handgrip strength and the presence of diabetic foot ulcers (ϕ = 0.190) and a rank-biserial correlation identified a significant positive relationship between low handgrip strength and Wagner scores (*r*_rb_ = 0.274, p = 0.011). Moreover, there was a moderate positive relationship between low handgrip strength and peripheral artery disease (ϕ = 0.312). However low handgrip strength was not significantly related with hypertension (ϕ = 0.095) and hyperlipidemia (ϕ = 0.124).

Also low handgrip strength was not significantly correlated with duration of diabetes (r = 0.130, p = 0.104), insulin treatment (ϕ = 0.023), HbA1c (r = −0.029, p = 0.724), serum creatinine (r = 0.011, p = 0.890), triglyceride level (r = 0.059, p = 0.495), LDL cholesterol (r = 0.003, p = 0.977) and e-GFR (r = 0.016, p = 0.846). But there were significant direct correlations between low handgrip strength and HDL cholesterol (r = 0.173, p = 0.047), age (r = 0.255, p = 0.001), and negative correlation with body mass index (r = −0.193, p = 0.018).

In the binary logistic regression analyses, model 1 was unadjusted; model 2 adjusted for sex and age; model 3 consisted of low handgrip strength, age, sex, hypertension, HbA1c, and model 4 consisted of low handgrip strength, age, sex, peripheral artery disease, HbA1c, and eGFR. Low handgrip strength was independently associated with diabetic foot disease in all models (Table 4).

## 4. Discussion

This prospective study has demonstrated that older patients with type 2 diabetes and diabetic foot disease have a higher rate of low handgrip strength than the older patients with type 2 diabetes who do not have diabetic foot disease. Moreover, low handgrip strength was significantly associated with the presence of diabetic foot ulcers in the analyses of correlation and regression. There is a previous study in the literature comparing these two groups of patients in terms of muscle strength and revealing similar results to this study [18]. This situation is predictable due to the increased inflammatory process in patients suffering from diabetic foot disease. Previous crosssectional studies have reported that low-grade chronic inflammation has a connection with age-related sarcopenia in the general populations [19] and grip strength has also been shown to be inversely related to systemic inflammation, fasting glucose, HBA1c, and hyperglycemia [20]. Grip strength is also related to multimorbidity load [21–23].

Furthermore, the presence of low handgrip was directly correlated with Wagner score in geriatric patients with type 2 diabetes in this study. Besides the direct correlation, patients with diabetic foot ulcers who had lower handgrip strength had significantly higher rates of Wagner scores 4 and 5 and lower rates of Wagner scores 1 and 3. There is a lack of information about the relationship between handgrip strength and the grade of diabetic foot disease in the literature. However, a recent study reported that patients with low grip strength were 50% less likely to have their wounds healed compared with those with adequate grip strength when corrected for other factors [16]. Instead of a direct causal relationship between grip strength and wound healing, it is more likely that grip strength reflects other variables that are probably reasons, such as nutritional status, and frailty [13–15].

Patients suffering from diabetic foot disease with normal and low muscle strength were also compared for factors that could affect muscle strength. Mean age, sex, and duration of diabetes were not significantly different between two groups. But the body weight and body mass index were significantly lower in patients with a diabetic foot ulcer. This result might be related to weight loss due to malnutrition and the inflammatory process. Moreover, the rates of hypertension, hyperlipidemia, ischemic heart disease, and the presence of complaints due to diabetic neuropathy were similar between these two groups. Also, the composition of oral antidiabetics, the rates of insulin treatment, duration of insulin treatment, and total daily insulin doses were similar between patients with diabetic foot ulcers who had low and normal handgrip strength.

The rate of statin use was higher in patients with normal grip strength than patients with lower grip strength. Against the fear of provoking myopathy, statin was researched about its effect on the physical functions of older patients. But the literature is inconsistent, as several studies could not reveal a decrease in functional performance or report any difference in muscle strength and exercise capacity [24, 25]. Moreover, some studies reveal similar results to this study. For instance, several studies suggested that statin use and exercise training had a positive interaction with the muscular response, performance, and proximal muscle strength [26–28]. The results of a recent study showed that statin intake moderated the effect of aerobic training on performance [29].

On the other hand, patients with diabetic foot ulcers who had lower handgrip strength had higher rates of peripheral artery disease than patients with diabetic foot ulcers who had normal handgrip strength. Moreover, peripheral artery disease and complaints of diabetic neuropathy were more common in patients with diabetic foot disease. Sarcopenia was previously reported as a prognostic factor for overall survival in patients with the peripheral artery disease [30]. In addition, studies also reported a positive association between preclinical atherosclerosis, handgrip, and gait speed [31, 32]. Moreover, another recent study revealed that sarcopenia was associated with the low ankle-brachial index, which is used in clinical settings for the detection of peripheral artery disease and sarcopenia could play an important role in the early detection of preclinical atherosclerosis [33]. The higher rate of low handgrip strength in patients with diabetic foot ulcers who had findings of peripheral artery disease seemed similar to the literature. The significant differences, especially the effect of peripheral artery disease on handgrip strength and sarcopenia, should be investigated with prospective studies in patients with diabetic foot disease.

The study is record-based; therefore, some data were missing for some patients like serum albumin and urine microalbumin levels. Moreover, this study is a crosssectional observational study and this design does not provide information about a causal relationship between diabetic foot disease and low handgrip strength or optimal handgrip strength value to predict the risk of diabetic foot disease. Prospective studies with a higher number of patients are needed to understand the role of handgrip strength and sarcopenia in the occurrence of diabetic foot ulcers. Also, the population included in this study is not appropriate to represent the general population. Because this study is performed in a tertiary diabetes care center, enrolling patients had longer diabetes periods and higher complication rates. There may be a possibility of selection bias in our study.

## 5. Conclusion

Geriatric patients with type 2 diabetes and diabetic foot disease have a higher rate of low handgrip strength than the patients with type 2 diabetes who do not have diabetic foot disease. Moreover, low handgrip strength was significantly associated with the occurrence of diabetic foot ulcers. Low handgrip was also directly correlated with Wagner score in older patients with type 2 diabetes. Considering the information obtained from this study, prospective studies with a higher number of patients are needed to fully understand the role of handgrip strength and sarcopenia in the occurrence of diabetic foot ulcers.

## Figures and Tables

**Table 1:** Demographics, clinical characteristics, biochemical results of the patients with type 2 diabetes according to existence of diabetic foot disease.

	Patients with type 2 diabetes who have diabetic foot ulcers n = 89	Patients with type 2 diabetes who do not have diabetic foot ulcers n = 69	p

Sex (Female) (n,%)	44 (49.4%)	45 (65.2%)	**0.047**

Age (year)	70.32 ± 4.70	69.89 ± 4.42	0.562

Duration of diabetes (year)	15.93 ± 6.67	13.86 ± 7.78	**0.046**

Diabetic retinopathy (%)	29.2%	17.4%	0.094

Complaints of neuropathy (%)	78 (87.6%)	47 (68.1%)	**0.005**

eGFR <60 mL/min/1.73 m2	36 (40.4%)	28 (40.6%)	0.987

Hypertension (n,%)	68 (76.4%)	58 (84.1%)	0.323

Hyperlipidemia (n,%)	61 (68.5%)	55 (79.7%)	0.163

Coronary artery disease (n,%)	42 (47.2%)	29 (42.0%)	0.518

Peripheral artery disease (%)	51.7%	6.7%	**0.004**

Smoking (n,%)	21 (24.1%)	10 (14.7%)	0.210

Body mass index (kg/m^2^)	31.23 ± 5.41	32.60 ± 6.54	0.163

Bodyweight (kg)	82.16 ± 14.45	81.23 ± 15.68	0.707

FPG (mg/dL)	237.24 ± 107.11	199.34 ± 106.47	**0.028**

HbA1c (%)	9.93 ± 2.04	9.26 ± 2.80	0.101

Triglycerides (mg/dL)	203.31 ± 115.56	214.52 ± 103.96	0.379

HDL cholesterol (mg/dL)	39.42 ± 12.57	42.16 ± 11.37	0.200

LDL cholesterol (mg/dL)	100.15 ± 35.24	108.54 ± 39.12	0.207

Non-HDL cholesterol (mg/dL)	136.60 ± 40.70	142.15 ±41.61	0.500

Creatinine (mg/dL)	1.12 ± 0.49	1.02 ± 0.32	0.127

e-GFR (mL/min/1.73 m^2^)	67.80 ± 29.32	65.40 ± 20.20	0.545

Uric acid (μg/L)	5.48 ± 1.77	5.71 ±1.56	0.094

Low handgrip strength	49 (55.1%)	25 (36.2%)	**0.019**

F: female; FPG: fasting plasma glucose; M: male; Wg: Wagner; eGFR: estimated glomerular filtration rate; LDL: low-density lipoprotein; HDL: high-density lipoprotein.

**Table 2:** The comparison of the drugs and insulin therapy according to existence of diabetic foot disease.

	Patients with type 2 diabetes who have diabetic foot ulcers n = 89	Patients with type 2 diabetes who do not have diabetic foot ulcers n = 69	p
**Metformin ** **(n,%)**	53 (59.6%)	47 (68.1%)	0.286
**Dpp-4 inhibitor ** **(n,%)**	40 (44.9%)	38 (55.0%)	0.145
**Sglt-2 inhibitor ** **(n,%)**	18 (20.2%)	12 (17.3%)	0.875
**Glp-1 analogue ** **(n,%)**	0 (0.0%)	1 (1.4%)	0.246
**Sulfonylurea ** **(n,%)**	14 (15.7%)	15 (21.7%)	0.395
**Thiazolidinedione ** **(n,%)**	7 (7.9%)	5 (7.2%)	1.000
**Glinid ** **(n,%)**	6 (6.7%)	7 (10.1%)	0.592
**Statin ** **(n,%)**	12 (13.4%)	22 (31.8%)	**0.007**
**Fenofibrate ** **(n,%)**	6 (6.7%)	10 (14,4%)	0.179
**Acetylsalicylic acid (n,%)**	59 (66.2%)	64 (55.0%)	0.154
**ACE inhibitor/ARB use (n,%)**	47 (52.8%)	47 (68.1%)	0.052
**Calcium channel blocker (n,%)**	30 (33.7%)	25 (36.2%)	0.871
**Beta blocker (n,%)**	28 (31.5%)	25 (36.2%)	0.645
**Insulin users (n,%)**	64 (71.9%)	34 (49.3%)	**0.004**
**Duration of insulin treatment (year)**	9.96 ± 4.89	8.92 ± 5.59	0.342
**Total daily insulin dose (U/day)**	44.43 ± 18.52	51.39 ± 31.54	0.249

**Table 3:** Demographic, clinical and laboratory parameters of type 2 diabetic patients with diabetic foot ulcer according to handgrip strength.

	Patients with low handgrip strength n = 49	Patients with normal handgrip strength n = 40	p
Age (year)	70.00 (11.00)*	75.00 (11.50)*	0.070**

Sex (F/M), (n)	22/27	22/18	0.462

Duration of diabetes (year)	20.00 (14.00)*	15.00 (10.00)*	0.611**

Bodyweight (kg)	80.00 (20.00)*	90.00 (16.30)*	**0.032** **

Body mass index (kg/m^2^)	26.42 (4.03)*	35.11 (11.92)*	**0.001** **

Hypertension (n,%)	37 (75.5%)	31 (77.5%)	1.000

Hyperlipidemia (n,%)	32 (65.3%)	29 (72.5%)	0.619

Ischemic heart disease (n,%)	24 (49.0%)	18 (45.0%)	0.872

Peripheral artery disease (%)	67.6%	29.2%	**0.009**

Diabetic retinopathy (n,%)	15 (30.6%)	11 (27.5%)	0.748

Smoking (n,%)	13 (26.5%)	8 (20.0%)	0.645

Systolic tension (mmHg)	125.00 (40.00)*	145.00 (40.00)*	0.163**

Diastolic tension (mmHg)	80.00 (20.00)*	80.00 (12.50)*	0.145**

FPG (mg/dL)	164.50 (194.30)*	166.00 (67.80)*	0.421**

Hba1c (%)	8.85 (4.13)*	9.91 (2.80)*	0.565**

Triglycerides (mg/dL)	180.50 (127.00)*	186.50 (165.50)*	0.267**

HDL cholesterol (mg/dL)	33.00 (10.90)*	37.35 (14.90)*	0.114**

LDL cholesterol (mg/dL)	98.25 (26.80)*	106.20 (46.90)*	0.648**

Non-HDL cholesterol (mg/dL)	123.40 (52.70)*	140.60 (78.40)*	0.191**

Urea (mg/dL)	39.25 (6.22)*	43.90 (18.15)*	0.177**

Creatinine (mg/dL)	0.91 (0.37)*	0.92 (0.39)*	0.837**

e-GFR (mL/min/1.73 m^2^)	71.98 (62.11)*	84.47 (25.47)*	0.869**

Uric acid (μg/L)	5.20 (3.30)*	5.25 (0.52)*	0.576**

Insulin users (n,%)	32 (65.3%)	32 (80.0%)	0.195**

Duration of insulin treatment (year)	10.00 (10.00)*	10.00 (6.00)*	0.504**

Total daily insulin dose (U/day)	40.00 (30.00)*	50.00 (31.00)*	0.355**

Wagner score	Wg 1= 4.2% Wg 2= 20.8% Wg 3= 35.9% Wg 4= 33.3% Wg 5= 6.3%	Wg1= 15.8% Wg2= 21.1% Wg3= 50.0% Wg4= 13.2% Wg5= 0.0%	**0.039**

F: female; FPG: fasting plasma glucose; M: male; Wg: Wagner; eGFR: estimated glomerular filtration rate; LDL: low-density lipoprotein; HDL: high-density lipoprotein.

* Median (IQR).

** Mann-Whitney U test was used to compare the median of continuous variables.

**Table 4:** Multivariate analyses of models.

Variable	Dependent variable	Independent variable	Odds ratio / 95% confidence interval	p value	R^2^ of the model	p value of the model
**Univariate**	Diabetic foot ulcer	Low handgrip strength	2.156 (1.131–4.115)	**0.019**	0.047	**0.019**
**Age and sex-adjusted**	Diabetic foot ulcer	Low handgrip strength	2.159 (1.100–4.255)	**0.025**	0.077	**0.026**
		Age	1.005 (0.933–1.082)	0.895		
		Sex	1.902 (0.984–3.675)	0.056		
**Model 1**	Diabetic foot ulcer	Low handgrip strength	2.066 (1.037–3.533)	**0.039**	0.109	**0.021**
		Age	0.992 (0.919–1.070)	0.827		
		Sex	2.005 (1.016–3.958)	**0.045**		
		Hypertension	0.876 (0.758–1.014)	0.566		
		HbA1c	1.563 (0.986–1.319)	0.076		
**Model 2**	Diabetic foot ulcer	Low handgrip strength	2.012 (1.004–4.032)	**0.049**	0.122	**0.038**
		Age	0.999 (0.924–1.080)	0.972		
		Sex	1.983 (0.998–3.940)	0.051		
		Hypertension	1.225 (0.513–2.923)	0.648		
		Hyperlipidemia	1.665 (0.735–3.768)	0.221		
		e-GFR (mL/min/1.73 m^2^)	0.998 (0.985–1.012)	0.824		
		HbA1c	0.864 (0.744–1.003)	0.054		

## Abbreviations

BB: Beta-blockers
DFU: diabetic foot ulcer
ACE inhibitor: angiotensin-converting enzyme inhibitors
ARB: angiotensin receptor blocker, CCB, calcium channel blockers
DPP-4i: dipeptidyl peptidase-4 inhibitor
SGLT-2i: sodium-glucose cotransporter-2 inhibitors
T2DM: type 2 diabetes mellitus
BMI: body mass index
HbA1c: glycosylated hemoglobin
FPG: fasting plasma glucose
Wg: Wagner
eGFR: estimated glomerular filtration rate
